# Pathological Analysis of Lateral Lymph Nodes in Patients With Advanced Rectal Cancer After Preoperative Chemoradiotherapy

**DOI:** 10.7759/cureus.103505

**Published:** 2026-02-12

**Authors:** Hiroshi Saito, Masanori Kotake, Hironori Hayashi, Kaeko Oyama, Takuo Hara

**Affiliations:** 1 Department of Surgery, JA Toyama Kouseiren Takaoka Hospital, Takaoka, JPN

**Keywords:** chemoradiotherapy, crt, lateral lymph node, lateral lymph node dissection, rectal cancer

## Abstract

Purposes: It remains unknown whether prophylactic excision of lateral pelvic lymph nodes, specifically the internal iliac and obturator lymph nodes, may be omitted after chemoradiotherapy (CRT) in rectal cancer patients without enlarged lateral lymph nodes (LLN). Therefore, we investigated the pathological outcomes of LLN dissection (LLND) for rectal cancer after preoperative CRT.

Methods: This retrospective single-center study aimed to determine whether LLND could be omitted after CRT in patients with advanced rectal cancer who had no LNN enlargement before treatment. Specifically, we evaluated the pathological positivity rate of dissected LLNs in patients who underwent LLND after CRT. Twenty-eight patients with rectal cancer who underwent LLND after preoperative CRT between April 2014 and February 2024 were included in the present study. They were stratified into the pretreatment LLN(+) (n=17) and pretreatment LLN(-) (n=11) groups based on multidetector-row computed tomography (MDCT) and magnetic resonance imaging (MRI) before treatment.

Results: The short diameter of LLN before pCRT was 10 (7-22) mm in the pretreatment LLN(+) group and 5 (2-6) mm in the pretreatment LLN(-) group. Pathological LLN metastasis was confirmed in eight (47%) patients in the pretreatment LLN(+) group (95% CI 26.2-69.0) and in none in the pretreatment LLN (-) group (95% CI 0-30.0) (p=0.01).

Conclusion: Pathological LLN metastasis was not identified in patients without radiological enlargement before treatment. These findings suggest that prophylactic LLND after preoperative CRT might be avoidable in carefully selected rectal cancer patients.

## Introduction

Preoperative chemoradiotherapy (CRT) and total mesorectal excision (TME) are widely performed globally for patients with locally advanced rectal cancer. Evidence from randomized trials indicates a greater reduction in local recurrence with preoperative CRT compared with either preoperative radiotherapy (RT) alone or postoperative chemoradiotherapy. [1-3]. The Japanese Society for Cancer of the Colon and Rectum (JSCCR) guidelines in 2022 recommend lateral lymph node dissection (LLND) for rectal cancer with cT3 or deeper staging, in which the lower edge of the tumor is located on the anal side of the peritoneal reflection. Although the rate of metastasis to the lateral lymph nodes (LLN) is reportedly approximately 15% [4], there are currently no established criteria for diagnosing LLN involvement, nor are there clear criteria for when it may be omitted. In recent years, however, some institutions in Japan have started to omit LLND in patients treated with preoperative CRT, indicating a gradual shift toward more selective approaches. Furthermore, it remains unknown whether prophylactic LLND after preoperative CRT may be omitted for advanced rectal cancer patients without enlarged LLN. Therefore, the present study examined the pathological outcomes of LLND for rectal cancer after preoperative CRT.

## Materials and methods

Study design and objectives

This was a retrospective single-center study. The aim of this study was to determine whether LLND could be omitted after CRT in patients with locally advanced rectal cancer who had no LLN enlargement before treatment. We evaluated the pathological positivity rate of LLNs in patients who underwent LLND after CRT at our institution.

Study population

Patients were eligible if they had advanced rectal cancer, received preoperative CRT, and subsequently underwent total mesorectal excision with LLND at our institution during the study period. Patients with unresectable stage IV disease were excluded. Between April 2014 and February 2024, 34 patients with advanced rectal cancer (clinical tumour 3-4 (cT3-4)) in our institution were treated with preoperative CRT followed by surgery, and 28 ultimately underwent LLND. They were stratified into the pretreatment LLN(+) (n=17) and pretreatment LLN(-) (n=11) groups based on diagnostic imaging before treatment.

Data collection

Clinical, radiological, operative, and pathological data were retrospectively collected from electronic medical records. Imaging findings were evaluated using multidetector-row computed tomography (MDCT) and magnetic resonance imaging (MRI).

Clinical staging and therapeutic strategy

Pretreatment clinical staging was determined using multi-detector row computed tomography (CT) and MRI. The position of the peritoneal reflection was evaluated by barium enema before preoperative CRT. Patients received 5-fluorouracil-based CRT with a total radiation dose of 50 Gy, in which the lateral pelvic region was included in the target volume. Definitive surgery was scheduled four to 14 weeks following completion of preoperative CRT. The size of the lateral pelvic lymph nodes was measured by MDCT, and the maximum long-axis and corresponding short-axis diameters were measured. Patients in whom the short diameter of the lateral lymph nodes was ≥7 mm in pre-CRT imaging were categorized as the pretreatment LLN(+) group, while those with diameters <7 mm were categorized as the pretreatment LLN(-) group. These values were selected based on previous studies that specified a cut-off diameter of 7 mm [5-7]. LLND was generally performed bilaterally. However, unilateral dissection was selected in cases considered to be at higher risk of metastasis on one side based on preoperative imaging findings, such as nodal enlargement or abnormal morphology. If the contralateral side showed no suspicious findings on imaging, dissection was not performed. In addition, unilateral dissection could be chosen when prolonged operative time was anticipated, at the discretion of the attending surgeon. Notably, among LLN-negative patients, unilateral dissection was performed in only one patient at the discretion of the attending surgeon.

Surgical procedure

All surgeries were performed by one surgeon (M.K.) with the endoscopic surgical skill qualification and robotic proctor proposed by the Japan Society for Endoscopic Surgery. LLND involved two parts: internal iliac node and obturator node dissection. After identification and mobilization of the ureteric fascia, the lateral side of the pelvic plexus was dissected. The autonomic nerves formed a continuous plane that represented the medial border of the internal lymph node dissection. The internal iliac lymph nodes, situated between the internal iliac artery and the autonomic nerves, were dissected along the vascular wall, starting from the bifurcation of the internal and external iliac arteries. During the obturator lymph node dissection, the plane between the internal iliac vessels and the obturator lymph nodes was developed and extended into the adipose tissue surrounding the urinary bladder. The lateral border of the obturator lymph node is defined as the external iliac vein, the iliopsoas muscle, and the internal obturator muscle. The obturator nerve was preserved, with concomitant resection of the obturator artery and vein.

Pathological analysis

A pathological analysis was conducted by multiple pathologists at our institution. Pathological assessment included lymph node yield, resection margin status, and metastatic status of harvested LLN, all of which may influence surgical strategy and oncological outcomes. The evaluation was primarily conducted through HE staining, while Elastica-van-Gieson staining was utilized to assess venous invasion and D2-40 staining for lymphatic invasion.

Statistical analysis

All statistical analyses were carried out using EZR software (Saitama Medical Center, Jichi Medical University, Saitama, Japan) [8]. Continuous data are expressed as median values with ranges, whereas categorical variables are reported as percentages. Differences between the pretreatment LLN(+) and LLN(-) groups were evaluated using the chi-square (χ²) test, Fisher’s exact test, or the Mann-Whitney U test, as appropriate. Statistical significance was defined as a p-value of less than 0.05.

## Results

**Figure 1:**
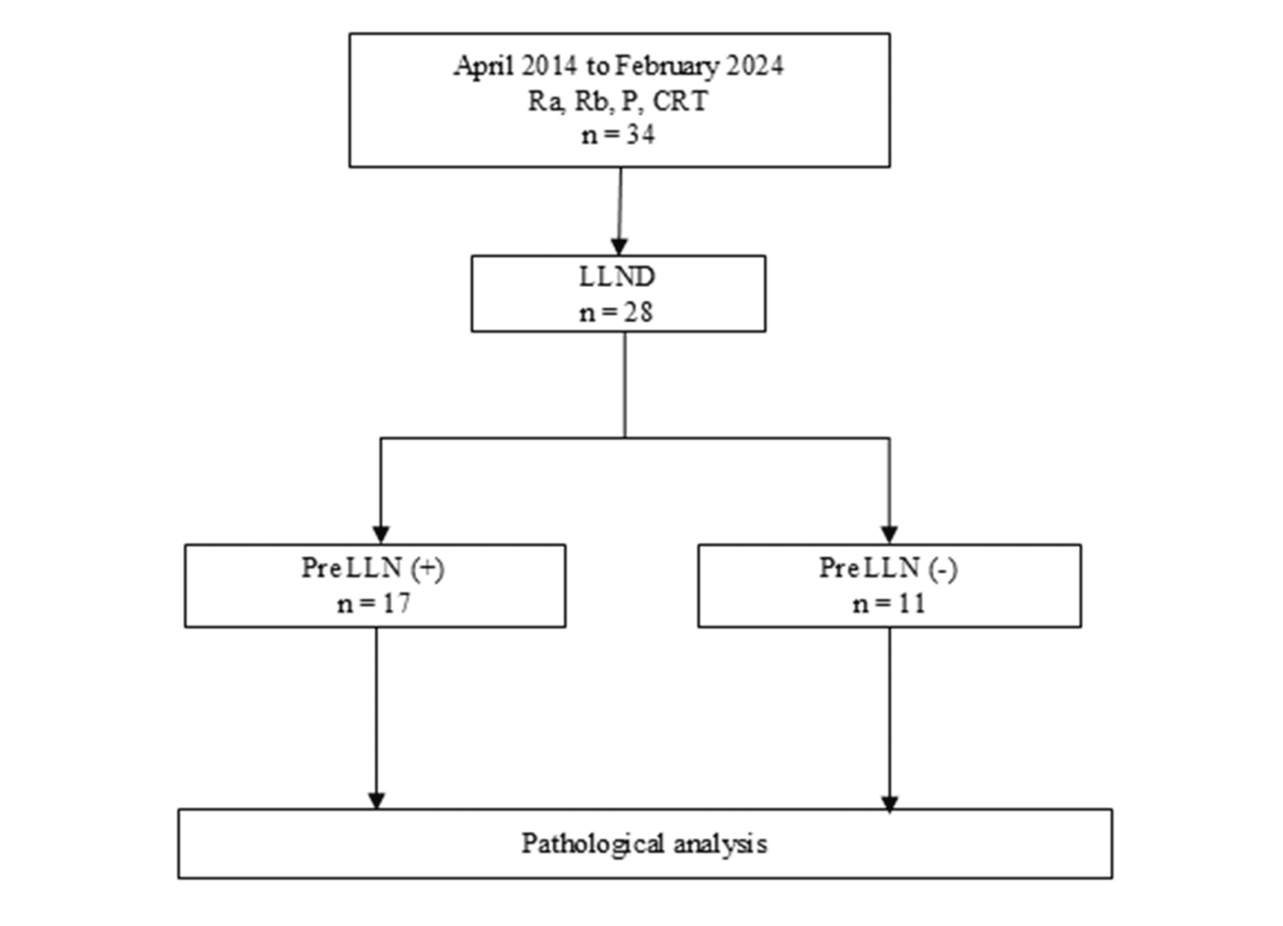
Flow diagram LLND: lateral lymph node dissection; LLN: lateral lymph nodes; Ra: upper rectum; Rb: lower rectum; P: anal canal; CRT: chemoradiotherapy

The study protocol is shown in Figure 1. Between April 2014 and February 2024, 34 patients received preoperative CRT for rectal cancer (cT3, upper rectum (Ra), lower rectum (Rb), and P) in our institution. Among them, 28 patients underwent LLND. They were stratified into the pretreatment LLN(+) (n=17) and pretreatment LLN(-) (n=11) groups based on the short diameter of LLN with pre-CRT MDCT.

Patient characteristics are summarized in Table 1. The percentage of male patients was significantly lower in the LLN(+) group (65 vs 100 %). The short diameter of LLN before CRT was significantly higher in the LLN(+) group (10 vs 5 mm). No significant differences were observed in age, BMI, American Society of Anesthesiologists (ASA) scores, clinical tumor (T) and nodes (N) stages, the CRT regimen, or the duration from the end of CRT to surgery between the two groups. The completion rate of CRT was 100% (28/28).

**Table 1:** Characteristics of patients who underwent LLND after pCRT LLN: lateral lymph nodes; ASA-PS: American Society of Anesthesiologists Physical Status Classification System; T: tumor; N: node; pCRT: preoperative chemoradiotherapy; LLND: lateral lymph node dissection; S-1: tegafur/gimeracil/oteracil; CapeOX: capecitabine+oxaliplatin; 5-FU/LV: 5-fluorouracil+leucovorin; FP: 5-fluorouracil+cisplatin

Variable	pre-LLN (+) (n = 17)	pre-LLN (-) (n = 11)	p-value
Sex, n (%)			0.0009*
Male	11 (65%)	11 (100%)	
Age (years)	67 (47-80)	68 (46-81)	0.981
BMI (kg/m2)	21.3 (16.1-28.8)	21.5 (17.9-28.1)	0.796
ASA-PS			1
1	1 (6%)	0 (0%)	
2	16 (94%)	11 (100%)	
Clinical T stage, n			1
T3	7 (41%)	4 (36%)	
T4	10 (59%)	7 (64%)	
Clinical N stage, n			0.0504
N0	0 (0%)	3 (27%)	
N1-3	17 (100%)	8 (73%)	
Largest short diameter of LLN before pCRT (mm)	10 (7-22)	5 (2-6)	<0.0005*
CRT regimen, n			1
S-1	13 (76%)	10 (91%)	
CapeOX	2 (12%)	1 (9%)	
5-FU/LV	1 (6%)	0 (0%)	
FP	1 (6%)	0 (0%)	
Duration from end of CRT to surgery (weeks)	10.9 (4.9-14.7)	10.3 (5.7-14.6)	0.888

Perioperative outcomes are summarized in Table 2. No significant differences were noted in the surgical procedure, blood loss, operative time, percentage of permanent stoma, or postoperative hospital stay between the two groups. We have been performing all rectum surgeries using robotic surgery since it was covered by the national health insurance system of Japan in 2019. The incidence of postoperative complications was slightly higher in the LLN(+) group (53 vs 18 %). Furthermore, the percentage of patients who received adjuvant treatments was slightly higher in the LLN(+) group (41 vs 19 %).

**Table 2:** Perioperative outcomes LLN: lateral lymph node; LLND: lateral lymph node dissection

Variable	pre-LLN (+) (n = 17)	pre-LLN (-) (n = 11)	p-value
Surgical method			0.435
Lap	9 (53%)	8 (73%)	
Robot	8 (47%)	3 (27%)	
Blood loss (ml)	20 (1-50)	30 (5-200)	0.402
Operative time (min)	570 (480-720)	570 (375-800)	0.906
Stoma, n (%)			1
diverting	7 (41%)	5 (45%)	
permanent	10 (59%)	6 (55%)	
LLND			0.619
Bilateral	13 (76%)	10 (91%)	
Unilateral	4 (24%)	1 (9%)	
Postoperative complications, n (%)			0.155
Overall complications	9 (53%)	2 (18%)	
Intraabdominal abscess	4 (24%)	0 (0%)	
Postoperative ileus	0 (0%)	2 (18%)	
Urinary dysfunction	4 (24%)	0 (0%)	
Surgical site infection	1 (6%)	0 (0%)	
Postoperative hospital stay (days)	19 (10-73)	16 (11-58)	1
Adjuvant treatment, n (%)	7 (41%)	2 (19%)	0.249

Pathological outcomes are summarized in Table 3. There were no significant differences in pathological TN factors or stages, CRT grades, or tumor differentiation between the two groups. The rates of lymphatic invasion and venous invasion were slightly higher in the LLN(+) group (44 vs 27%, 47 vs 27%). Pathological LLN metastasis was confirmed in eight (47%) patients in the LLN(+) group (95% CI 26.2-69.0) and in none in the LLN (-) group (95% CI 0-30.0) (p=0.01). However, nine patients in the LLN(+) group did not have metastasis to the LLN, and the size of the LLN was reduced in all cases after neoadjuvant CRT. Therefore, viable cancer cells were considered to originally be present in the LLN in these cases but were eliminated by neoadjuvant CRT.

**Table 3:** Pathological outcomes LLN: lateral lymph nodes; T: tumor; N: node; CRT: chemoradiotherapy

Variable	pre-LLN (+) (n = 17)	pre-LLN (-) (n = 11)	p-value
Pathological T stage, n			1
CR	3 (18%)	2 (18%)	
T1-2	2 (12%)	1 (9%)	
T3-4	12 (70%)	8 (73%)	
Pathological N stage, n			0.253
N0	8 (47%)	8 (73%)	
N1-3	9 (53%)	3 (27%)	
LLN metastasis rate, n (%)	8 (47%)	0 (0%)	0.01*
Pathological stage, n			0.262
CR	2 (12%)	2 (18%)	
1-2	4 (24%)	6 (55%)	
3	5 (29%)	2 (18%)	
4	6 (35%)	1 (9%)	
CRT grade, n			0.874
1	7 (41%)	3 (27%)	
2	7 (41%)	6 (55%)	
3	3 (18%)	2 (18%)	
Tumor differentiation, n			1
Well or moderately differentiated	15 (88%)	10 (91%)	
Poorly differentiated/mucinous carcinoma	2 (12%)	1 (9%)	
Lymphatic invasion, n (%)	7 (44%)	3 (27%)	0.689
Venous invasion, n (%)	8 (47%)	3 (27%)	0.435

## Discussion

In Japan, a number of studies have examined CRT and LLND for rectal cancer patients. The Japan Clinical Oncology Group Study 0212 (JCOG0212) study demonstrated that the local recurrence rate was lower with the addition of LLND for lower rectal cancer than with TME alone [9]. Kuster et al. reported that the local recurrence rate for advanced lower rectal cancer with TME alone was 12% [10]. In Western countries, preoperative CRT has become the standard treatment for locally advanced low-lying rectal cancer [11, 12]. On the other hand, the JSCCR guidelines recommend TME and LLND as the standard treatment for advanced lower rectal cancer [13]. However, some facilities in Japan have also adopted preoperative CRT and TME as the standard treatment in recent years [14]. A phase III trial to compare the treatment outcomes of preoperative RT and prophylactic LLND has yet to be conducted. However, Nagawa et al. reported the findings of a small-scale comparative trial in which patients were randomly assigned to either the CRT followed by TME group or the TME+LLND group. No significant differences were observed in overall survival, disease-free survival, or the local recurrence rate between the two groups [15]. Watanabe et al. conducted a study on 115 patients who underwent surgery for rectal cancer. They compared four groups based on the presence or absence of RT and lateral LLND in addition to TME: “RT+, LLND-“, “RT+, LLND+”, “RT-, LLND-“, and “RT-, LLND+”, and found that disease-free survival rates did not significantly differ between the “RT+, LLND-” and “RT-, LLND+” groups [16]. These findings and the present results indicate that preoperative CRT and prophylactic LLND are equivalent as additional treatments to TME. However, it is important to note that based on the results of this study, this may not apply when enlarged LLNs are detected before CRT. In other words, therapeutic LLND may still be necessary even after preoperative CRT.

Previous studies examined the size of LLN [5-7, 17]. In a multinational study involving 12 institutions, Ogura et al. established a cut-off value of 7 mm for LLN based on findings showing that patients with LLN >7 mm had more advanced clinical stages. In cases with LLN ≥7 mm, the local recurrence rate after preoperative CRT+TME was 19.5%, while it was significantly lower at 5.7% in cases in which CRT+TME+LLND was performed [5]. Other studies from Japan selectively performed LLND after CRT for cases with LLN ≥7 mm and reported excellent disease-free survival rates [6, 7]. Therefore, a cut-off value of 7 mm was set for the short-axis diameter in the present study. Based on an accurate LLN diagnosis, prophylactic LLND may be unnecessary after preoperative CRT followed by TME in cases in which the clinical diagnosis of LLN metastasis is negative.

Although the impact of LLND on locoregional recurrence and overall survival remains a matter of debate, particularly in Western countries where this procedure is not routinely performed, these outcomes could not be adequately assessed in the present study because of its retrospective design and limited follow-up. Nevertheless, our pathological findings demonstrated a substantial incidence of metastasis in LLNs that were enlarged before CRT. From a practical standpoint, these results may provide useful information for surgical decision-making and suggest that LLND should still be considered in patients with suspicious nodes prior to treatment. Future prospective investigations evaluating long-term oncological outcomes will be required to clarify the survival benefit of this strategy.

There are several limitations that need to be addressed. First, because the extent of LLND was determined based on preoperative imaging and intraoperative judgment, occult metastasis in non-dissected nodes cannot be completely excluded. Therefore, the true incidence of LLN metastasis may have been underestimated. However, unilateral dissection in radiologically negative cases was rare in our cohort, and most patients underwent bilateral dissection, which may have minimized this potential bias. In addition, this was a retrospective single-center analysis. Moreover, the sample size was relatively small, which limited the statistical power of the analysis and precluded robust subgroup evaluations. Therefore, the generalizability of our findings should be interpreted with caution. Larger, preferably multicenter studies will be necessary to validate the present results. Another limitation is the large number of pathologists involved, which may have led to various opinions and, as a result, different interpretations.

## Conclusions

The present study demonstrated that pathological metastasis after CRT was not identified in patients with advanced rectal cancer whose LLNs were not enlarged before treatment. Based on these findings, preoperative CRT for radiologically negative LLNs may represent a reasonable alternative strategy to routine LLND in carefully selected patients. In contrast, when LLNs were enlarged prior to treatment, a considerable rate of residual metastasis remained even after CRT, suggesting that LLND should still be considered in this population. Given the retrospective design and limited sample size, further validation in larger, preferably multicenter studies will be required to confirm these observations.
